# MetaPaCS: A Novel Meta-Learning Framework for Pancreatic Cancer Subtype Identification

**DOI:** 10.1002/aisy.70576

**Published:** 2026-10-15

**Authors:** Nick B. Peterson, Mengtao Sun, Xinchao Wu, Jieqiong Wang, Shibiao Wan

**Affiliations:** 1 Department of Genetics, Cell Biology and Anatomy, University of Nebraska Medical Center, Omaha, Nebraska, USA; 2 Department of Neurological Sciences, University of Nebraska Medical Center, Omaha, Nebraska, USA; 3 Fred and Pamela Buffett Cancer Center, University of Nebraska Medical Center, Omaha, Nebraska, USA

**Keywords:** bioinformatics, computational biology, pancreatic cancer, risk stratification, subtyping

## Abstract

Pancreatic cancer (PaC), the third leading cause of cancer related deaths in the United States, is a highly heterogenous malignancy with a multitude of potential subtypes. Each PaC subtype has unique molecular pathways and therapeutic characteristics, which can be used in downstream patient risk stratification and tailored treatment design. Conventional wet-lab approaches for PaC subtyping (microdissection, histopathological studies, etc.) are laborious, costly, and time-consuming. To address these concerns, we present MetaPaCS, a novel meta-learning framework to accurately identify PaC subtypes based on transcriptomics data. Specifically, after preprocessing, the transcriptome-based feature vectors are classified by multiple base machine learning classifiers, whose diverse prediction outputs are combined with the initial preprocessed feature vectors to constitute a new set of ensemble feature vectors for a meta-learning classifier. Leave-one-out cross-validation demonstrates that MetaPaCS performs remarkably better optimized individual base classifiers. We believe that MetaPaCS is a promising tool for downstream risk stratification and personalized treatment design.

## 1 | Introduction

Pancreatic cancer (PaC), a highly lethal malignancy that is nearly undetectable in its earlier stages, is the third leading cause of death in the USA and is projected to become the second within a decade [1, 2]. Since 1970, the amount of new PaC cases per year has increased drastically from 18 800 to 64 050 in 2023 [3]. In contrast, PaC’s 5-year survival rate has only improved by 3% in that same time frame, going from a 9% 5-year survival rate to a 12% 5-year survival rate, while breast cancer’s 5-year survival rate has improved from 40%–50% in 1970 to 90%–100% in 2023 in that same time frame [4]. Additionally, PaC’s mutational landscape is highly heterogenous among patients and within the tumor itself, making it difficult to find a singular effective treatment for all PaC cases. While current more generalized treatments like neoadjuvant therapy have resulted in slight improvements, the most promising improvements have instead come from experimental targeted treatments like immunotherapy using novel delivery methods [5]. The effectiveness of these new personalized treatment options highlights the benefits of researching the variation that can be found in PaC’s different molecular pathways.

Subtyping has become an increasingly popular way to provide more personalized healthcare. A notable example is the improved prognostic and predictive performance for breast cancer when the subtype information is incorporated into diagnosis [6]. An initial study in 2015 found two distinct subtypes of PaC, namely basal-like and classical, though even within these subtypes there is high variation between each individual case [7]. Further PaC subtyping has been conducted via expression analysis correlating with previously seen histopathological characteristics from microdissection, identifying four key subtypes of PaC: aberrantly differentiated endocrine exocrine (ADEX), immunogenic, progenitor, and squamous [1]. Each subtype shows differing characteristics in treatment and survival [1]. Specifically, the immunogenic subtype is associated with upregulation in genes involved with B-cell signaling pathways, antigen presentation, CD4+ T-cell signaling, CD8+ T-cell signaling, and Toll-like receptor signaling pathways, creating an immunosuppressant microenvironment. As a result, this subtype would likely respond less to immunotherapy. The squamous subtype shows mutations similar to those seen in other C2-squamous-like class tumors of different cancers and may share therapeutic strategies. These unique characteristics ultimately make subtyping each PaC case a logical choice for both creating the most efficient individual treatment plans and for minimizing the side effects of the more general treatments.

Conventionally, wet-lab-based approaches are used for identifying PaC subtypes, with the subtypes defined histopathologically being found through microdissection and examination of the samples under a microscope, which is labor intensive, requires the resection of the tumor, and may be infeasible for the patients where tumor resection is not possible [7]. Biomarker-based methods are becoming another increasingly used type of wet-lab approach for subtyping other cancers, though they are currently not suitable for PaC subtyping [8, 9]. Ca19–9, the only FDA-approved biomarker for PaC, has a low sensitivity and is not subtype specific, leading to late diagnoses [3]. In addition, Ca19–9 is ineffective for diagnosis in the 5%–10% of people who possess the Lewis negative phenotype due to the reduced synthesis of Ca19–9 [10]. While other promising biomarkers are currently being researched for diagnosis such as MIC, TNP1, and RIT2, they are still in the early stages of testing and are unable to differentiate between subtypes [3]. In summary, current wet-lab methods to characterize PaC subtyping are time-consuming, labor-intensive, expensive, or ineffective.

Computational methods offer an alternative solution for efficient and cost-effective PaC subtyping, as the omics data of a tumor is much less invasive to obtain and does not require the presence of large tumors for analysis. Computational methods like machine learning (ML) approaches have been widely applied to different aspects of cancer studies [11, 12], including risk stratification prediction [13, 14], subtype characterization [15, 16], and subtype prediction [17]. Due to their versatility and ability to handle large amounts of data, computational methods can make full use of features that may otherwise be difficult for a human researcher, including clinical data, omics data [18], image data [19], or multimodal data. Specific methods for PaC subtype prediction include the use of nonnegative matrix factorization-derived meta-genes in H_2_O [20]. Another study makes use of multiple deep learning models, including Vanilla, IDaRS, DeepMIL, and VarMIL, to predict PaC subtype based on whole slide pathological images [21].

While these ML approaches have demonstrated different levels of competitiveness for PaC subtyping, they may not perform robustly and consistently across different datasets and cases due to limited generalizability. Stacking is an ensemble learning technique which uses multiple different classifiers in its prediction, preventing the model from overfitting and reducing the overall influence of bias, thus enhancing the model’s generalization capabilities [22]. A stacking model makes use of multiple base classifiers with different strengths and characteristics to generate a set of initial prediction probabilities, which can then be used as input data for a meta-learning classifier. This allows the meta-learning classifier to learn from the base classifier’s biases and account for them, improving the robustness of the results [23]. Similar models have shown high performance for other biological tasks like Alzheimer’s diagnosis [24], lung cancer characterization [25], and breast cancer in past studies [22].

To this end, we propose a novel meta-learning approach, MetaPaCS, for the accurate and reliable prediction of PaC subtypes based on transcriptomics data only. Transcriptomics data-based ML models have been successfully used in the subtyping of multiple other cancers such as B-cell acute lymphoblastic leukemia [26–28], ovarian cancer [29], and medulloblastoma [28], due to their ability to provide a direct look at molecular pathway activity within a tumor. MetaPaCS makes use of multiple base classifier algorithms in a stacking model for both a more robust classification scheme and a reduced risk of algorithmic bias. We demonstrate that MetaPaCS can both outperform any individual ML classification method in subtype classification tasks and is shown to improve the visualization of PaC subtypes when compared to methods like PCA, UMAP, and t-SNE.

## 2 | Methods

### 2.1 | Dataset Collection and Preprocessing

In this study, we used the RNA-seq expression data from 96 PaC patients previously reported by Bailey et al. [1]. These samples, all between 34 and 90 years of age, were classified into four different subtypes (Figure 1A,B). To prevent the possible skewing of results, missing values in the expression data were replaced with the average expression value for that same gene across all samples using imputation. The data were then converted back into counts per million (CPM) before the coefficient of variation squared (CV^2^) was calculated for each gene. Only genes with a CPM above 1 and a CV^2^ above 0.5 were kept, reducing the number of genes from 18 276 to 367, preserving only the most varied genes for classification (Figure 1C).

### 2.2 | Meta Learning for Pancreatic Cancer Subtyping

For PaC subtyping, we proposed MetaPaCS, a novel meta-learning framework for accurate identification of PaC subtypes based on transcriptomic data only. Our model was made up of 2 main blocks, Block A, containing the selected base classifiers, and Block B, containing the meta-learner (Figure 1D). A pool of 10 ML algorithms was evaluated during framework optimization. For each candidate framework, a selected subset of these 10 algorithms was used in Block A to generate subtype probability outputs, which were concatenated with the original input data and supplied to a candidate Block-B classifier.

Ten ML algorithms were considered as candidate classifiers during framework optimization: support vector machines (SVMs) with either a linear kernel (SVM linear) [30] or a radial basis function kernel (SVM RBF) [31], decision tree classifier (DT) [32], random forest classifier (RF) [33], XGBoost (XGB) [34], Gaussian naïve Bayes (NB) [35], logistic regression (LR) [36], multilayer perceptron (MLP) [37], K-nearest neighbors (KNN) [38], and quadratic discriminant analysis (QDA) [39]. These classifiers were chosen due to their diverse methods of analysis, ensuring a variety of predictions for a more holistic predictive performance [40]. To better enhance the performance of the base classifiers and quality of predictions, some of the possible classifiers chosen were ensemble methods themselves. All ML classifiers aside from XGB were implemented using SKlearn [41]. XGB was implemented using the Python XGBoost package [42].

MetaPaCS was designed as a flexible two-block stacking framework. For each candidate framework, a subset *S*containing *M* base classifiers were selected from the ten candidate algorithms for Block A. The subtype probability outputs generated by the selected base classifiers were concatenated with the original filtered expression features to construct an augmented input representation for Block B. During framework evaluation, Block-A subsets containing different numbers and combinations of candidate classifiers were examined, and each of the ten candidate algorithms was also evaluated as the Block-B meta-learner. Model performance was assessed using prediction accuracy, where the best-performing framework was adopted as the final MetaPaCS framework. Detailed hyperparameters for all classifiers evaluated in Blocks A and B are shown in Table S1. To ensure that the probability features supplied to Block B were generated out-of-fold, each selected Block-A classifier was evaluated using leave-one-out cross-validation (LOOCV). For each sample *i* and each base classifier *m* ∈ S, the classifier was fitted using the remaining samples, denoted as *D*_−*i*_, which was then used to calculate a four-class subtype probability vector exclusively for the held-out sample

$$p_{i}^{\left(m\right)}=f_{-i}^{\left(m\right)}\left(x_{i}\right),m\in S$$

where $f_{-i}^{\left(m\right)}$ denotes base classifier *m* trained without sample *i*, and $p_{i}^{\left(m\right)}$ contains the predicted probabilities for the four PaC subtypes. Repeating this procedure for all samples generated out-of-fold probability matrix for each selected base classifier. For a framework containing *M* base classifiers, the augmented representation for sample *i* was constructed as

$$z_{i}=\left[x_{i},p_{i}^{\left(m_{1}\right)},p_{i}^{\left(m_{2}\right)},\ldots ,p_{i}^{\left(m_{M}\right)}\right]$$

where ***x***_*i*_ represents the filtered expression features of sample *i*.

LOOCV was used to evaluate the performance of MetaPaCS and other state-of-the-art approaches for PaC subtyping. Nine different metrics were used to provide a more comprehensive evaluation of a model’s effectiveness: accuracy, area under the curve (AUC), Matthews correlation coefficient (MCC), *F*1-score, recall, *G*-measure, specificity, Jaccard index, and precision.

### 2.3 | Model Testing and Optimization

To optimize our framework, we tested multiple combinations of base and meta-learning classifiers to better analyze the learning process and find the highest performing classifier combinations for each block. First, different numbers of base classifiers were tested to see if the additional prediction data would improve the meta-learning performance. Block-A models, including two, four, six, eight, and ten of the most accurate base classifiers, were evaluated for all meta-learning classifiers in Block B for comparison (Figure S1). Additionally, we tested the effects of different classifier combinations on the meta-learning performance of the model by evaluating the model’s performance on all unique combinations of two from the 10 possible classifiers in Block A, with the model performance being evaluated for all 10 possible meta-learning classifiers in Block B (Figure S2).

Other additional classifiers were used due to factors such as relatively low computational burden and good performance. KNN, LR, NB, and DT were chosen due to their high computational efficiency [43, 44]. QDA and MLP were chosen due to their relatively high performance, with QDA being highly effective on classes with different covariances and MLP being able to iteratively learn data patterns with backpropagation [45]. Both linear and radial basis function SVMs were used for their strong performance in high-dimensional data, which was especially useful in the high-dimensional RNA seq data.

### 2.4 | MetaPaCS Visualization

To capture both the global structure of the data and the specificity of subtype predictions for MetaPaCS, we made use of UMAP, t-SNE, and PCA using prcomp, the Rtsne, and uwot packages to evaluate the effectiveness of MetaPaCS at visualization compared to basic visualization methods. Specifically, MetaPaCS utilized a combination of two key matrices: a subtype score matrix and a sample-to-subtype matrix derived from prediction results. The former matrix was obtained through the MetaPaCS subtype identification workflow (Figure 1), in which subtype-specific scores were calculated for each sample. This matrix captured the continuous prediction confidence of each sample across the four PaC subtypes, forming a *S* × *L* matrix, where *S* is the number of samples and *L* is the number of subtypes. A sample-to-subtype matrix was constructed using one-hot encoding based on the predicted subtype labels derived from the subtype score matrix. Specifically, the subtype with the highest score for a given sample was assigned a value of 1, while all other subtypes were assigned 0. This resulted in a binary matrix of the same dimension (*S* × *L*), representing discrete subtype assignments. These two matrices were then concatenated along the feature axis to form a unified visualization matrix, which was used as input for t-SNE, PCA, and UMAP to generate a low-dimensional embedding. This integration strategy enabled the combination of both quantitative prediction certainty and categorical class identity, enhancing the biological interpretability and subtype-level separation in the visualization space.

### 2.5 | Subtype-Specific Differential Gene Expression Analysis

Subtype-specific differential gene expression (DGE) analysis was conducted via one-versus-rest comparisons for each subtype. Genes were considered subtype specific if they met criteria of *p*-value < 0.05 and log2 Fold Change (logFC) > 1. The 10 most differentially upregulated genes from each subtype were then visualized in a heatmap to highlight the transcriptional difference across subtypes. Additionally, volcano plots were generated for each subtype with a threshold of |log2FC| > 1.5 and adjusted *p*-value < 0.05. Finally, t-SNE plots were generated based on differential expression profiles, with samples colored by the expression of a significantly up-regulated gene corresponding to a specific subtype.

### 2.6 | Gene Ontology (GO) Pathway Analysis

The top 500 upregulated genes for each subtype were subjected in Gene Ontology (GO) using ClusterProfiler (V3.21) enrichment analysis to identify the top 15 most significantly enriched pathways within biological processes, cellular components, and molecular function [46].

### 2.7 | Gene–Drug–Disease Association Studies

Drug and disease linkages were extracted from the DrugBank database [47], and the target–disease linkages were curated in the Therapeutic Target Database (TTD) [48]. The upand downregulated genes were cross-referenced against these pharmacological datasets to give a list of drugs for both possible novel therapeutic agents for each cancer subtype and potential effects each subtype may have on the metabolism and function of these existing agents.

### 2.8 | Statistical Analysis

For statistical analysis, DGE analysis was performed using one-versus-rest comparisons with two-sided empirical Bayes-moderated *t*-tests implemented in the *limma* package. *P*-values were adjusted using the Benjamini–Hochberg method, and adjusted *P* < 0.05 was considered statistically significant. GO enrichment analysis was performed using *clusterProfiler* package with adjusted *P* < 0.05. Analyses were conducted using R 4.4.1.

## 3 | Results

### 3.1 | Model Optimization and Benchmarking for Identifying PaC Subtypes

To determine the optimal framework of MetaPaCS, a wide range of classifier combinations for Block A and Block B were systematically evaluated. Each of the ten base classifiers in Block A was first trained individually and ranked according to performance to provide a reference for model selection. Subsequently, the influence of different base-classifier combinations on meta-learning performance was assessed. Block A was constructed using the top two, four, six, eight, or all 10 classifiers, and each configuration was exhaustively paired with 10 candidate meta-learners in Block B (Figure S1). Additionally, all possible pairs of two base classifiers were tested from the 10 possible base classifiers to evaluate the impact of each individual base classifier on the performance of Block B. This comprehensive search enabled evaluation of how classifier diversity and ensemble breadth affected overall predictive performance (Figure S2). Among the 147 evaluated configurations, KNN and QDA as the base learners combined with XGBoost as the meta-learner ranked first for eight of the nine evaluation metrics and achieved the best overall average rank (Figure S3). Based on its most robust and accurate subtype predictions across accuracy, precision, sensitivity, *F*1-score, MCC, *G*-measure, AUC, specificity, and Jaccard index, this configuration was selected as the final MetaPaCS framework. To assess the robustness of the framework selection results, all 147 candidate combinations were further evaluated using nested fivefold cross-validation. The best-performing alternative framework achieved an accuracy of 0.9063, which did not exceed the accuracy of 0.9167 obtained by the selected KNN–QDA/XGBoost MetaPaCS framework (Figure S4). Therefore, the original MetaPaCS configuration was retained for the final framework. To further benchmark MetaPaCS against simple ensemble strategies, probability averaging and majority voting were implemented using KNN, QDA, and XGBoost. MetaPaCS achieved higher accuracy (0.9167), *F*1-score (0.9172), and MCC (0.8876) than probability averaging (0.8958, 0.8960, and 0.8615, respectively) and majority voting (0.9063, 0.9066, and 0.8747, respectively). MetaPaCS also showed higher sensitivity, G-measure, specificity, and Jaccard index. However, probability averaging achieved the highest AUC (0.9916), compared with 0.9801 for MetaPaCS and 0.9757 for majority voting (Figure S5).

The predictive performance of the optimized MetaPaCS framework was benchmarked against each of the ten independently trained base classifiers (Figure 2). These individual classifiers were first trained and performance-ranked to establish a comparative reference. Across all evaluated metrics, MetaPaCS consistently achieved the highest performance, outperforming all single-model classifiers in all performance metrics (Figure2A–I). These findings highlighted the advantage of the meta-learning framework, which could integrate complementary information from diverse base learners to produce more stable and accurate subtype predictions compared with individual classifiers.

### 3.2 | Subtype-Specific Differential Gene Expression Analysis Showed Distinct Patterns of Expression Separating the PaC Subtypes

To assess the impact of MetaPaCS on visualizing PaC subtypes, three commonly used dimensionality reduction and visualization methods, including principal component analysis (PCA), t-distributed stochastic neighbor embedding (t-SNE), and uniform manifold approximation and projection (UMAP), were applied to different feature representations (Figure 3). When these methods were applied directly to the raw gene expression matrix (Figure 3A–C), samples from the four subtypes (squamous, ADEX, progenitor, and immunogenic) showed extensive overlap, and clear subtype boundaries were not apparent. This pattern indicated that conventional unsupervised visualizations based solely on expression profiles were insufficient to resolve the underlying subtype structure. In contrast, substantially improved clustering was observed when the same dimensionality reduction methods were applied to the MetaPaCS-derived embedding (Figure 3D–F), where samples formed more compact and well-separated clusters, reflecting that MetaPaCS captures discriminative structure that was not evident in the original expression space. Visualization was further enhanced when the MetaPaCS embedding was concatenated with one-hot-encoded (OH) subtype probabilities before dimensionality reduction (Figure 3G–I), resulting in tightly clustered groups with minimal inter-subtype overlap. Among the three visualization methods, t-SNE applied to the MetaPaCS-based representation produced the most distinct separation of subtype clusters, consistent with its emphasis on preserving local neighborhood structure. These findings indicated that MetaPaCS provided a more informative, subtype-aware feature space that markedly improved the effectiveness of standard visualization techniques, with t-SNE being selected as the primary method for subsequent graphical representation of subtype structure.

### 3.3 | Subtype-Specific Differential Gene Expression Analysis Showed Distinct Patterns of Expression and Potential Biomarkers for PaC Subtypes

To delineate subtype-specific expression patterns and potential biomarkers for PaC, DGE analysis was performed (Figure 4). As shown in the heatmap and volcano plots (Figure 4A–E), the four subtypes exhibited distinct signatures, with sets of genes selectively and significantly up- or downregulated in each subtype. These subtype-specific patterns highlighted underlying biological differences and revealed potential biomarkers associated with their clinical characteristics. Progenitor displayed significant upregulation of G6PC2, a member of the glucose-6-phosphatase catalytic subunit family that participate in the final steps of gluconeogenic and glycogenolytic pathways. Although G6PC2 lacks phosphohydrolase activity, it is selectively expressed in pancreatic islets and plays a critical role in modulating basal glucose homeostasis through the regulation of glucose-6-phosphate handling. Dysregulation of G6PC2 has been associated with alterations in fasting blood glucose levels and pancreatic β-cell function, suggesting that its elevated expression in the progenitor subtype may reflect subtype-specific metabolic reprogramming consistent with pancreatic lineage features [28]. Immunogenic showed a high amount of upregulated immunoglobin genes such as IGKV1.39 and LGKV6D.21 [49]. The squamous subtype exhibited elevated expression of epithelial and migration-related markers, including MIR205HG and KRT6C. MIR205HG, the host transcript of miR-205, has been linked to epithelial differentiation and enhanced invasive potential in squamous-associated malignancies. KRT6C, a cytokeratin enriched in proliferative squamous epithelia, has been shown to drive tumor cell proliferation, migration, and epithelial–mesenchymal transition activation, and its high expression correlates with poor prognosis and aggressive behavior in epithelial cancers such as lung adenocarcinoma [50, 51]. The ADEX subtype exhibited marked upregulation of pancreas-enriched digestive enzymes, including CLPS and CELA3B. CLPS encodes colipase, an essential cofactor for pancreatic lipase and a key component of the exocrine digestive system, reflecting strong pancreatic lineage identity. CELA3B, which is exclusively expressed in the exocrine pancreas, encodes a chymotrypsin-like elastase whose dysregulation has been mechanistically linked to chronic pancreatitis, β-cell dysfunction, and increased susceptibility to pancreatic adenocarcinoma, as demonstrated in both human genetic studies and CRISPR-engineered mouse models [52]. Additionally, the expression level of upregulated DEGs, including *G6PC2*, *IGKV1.39, MIR205HG*, and *CLPS*, across all 96 samples highlighted their specific overexpression in progenitor, immunogenic, squamous and ADEX, respectively (Figure 4F–I).

### 3.4 | Subtype-Specific Pathway Analysis Identified Distinct Enriched Pathways for PaC Subtypes

The difference between subtypes was further examined through the 15 most prevalent GO pathways of the top 500 upregulated genes in each (Figure 5). The progenitor subtype (Figure 5A) mainly displayed upregulated cellular transport and secretion-associated genes, with the most notable upregulated gene being insulin secretion. The squamous subtype (Figure 5B) showed a high upregulation in both internal and external cellular structure-related pathways such as epidermis development and intermediate filament organization. Additionally, four of the 15 upregulated pathways in the squamous subtype were involved in kidney cell differentiation. The immunogenic subtype’s (Figure 5C) most upregulated pathways were nearly all immunity pathways, with the only exception being the 17 genes involved in the digestion pathway. The ADEX subtype’s (Figure 5D) most upregulated pathways were mostly based on the secretion of different peptides and hormones, with only three pathways based on transport and two based on localization.

### 3.5 | Drug–Disease Association Studies Identified Potential Subtype-Specific Target Genes and Drugs

Pathway analysis was then conducted on these differentially expressed genes for associated drugs and diseases using the drugbank database (Figure 6). We found that many of these subtype-specific upregulated genes were linked to a multitude of drugs and diseases. In the progenitor subtype (Figure 6A), for example, the upregulated gene NR1I2 was linked to the metabolism of halothane, a commonly used anesthetic in developing countries that is known for severe hepatotoxic side effects [53]. The squamous subtype (Figure 6B) showed upregulation in the gene ANXA1, which impacted the metabolism of a multitude of drugs that treated everything from autoimmune issues like arthritis to infectious disease like COVID-19. TNFRSF17, which was associated with the metabolism of five different medicines that were used for the treatment of multiple myeloma, was found to be upregulated in the immunogenic subtype (Figure 6C). The upregulated genes of the ADEX subtype (Figure 6D) showed interactions with 12 different drugs, with several being associated with metabolism, digestion, and two forms of hematological cancer. Additionally, the gene AMY2A, responsible for starch digestion, was notably upregulated in the ADEX subtype.

## 4 | Discussion

MetaPaCS is a novel stacking-based framework for predicting PaC subtypes using RNA-seq data. Given the high complexity and heterogeneity of PaC transcriptomes, no single algorithm is universally suited for this classification task, and model performance is inevitably influenced by algorithmic bias. For example, linear SVM assumes linear separability and therefore cannot accurately estimate decision boundaries when the underlying relationships are nonlinear [54]. Moreover, a comprehensive benchmark evaluating 179 algorithms across 121 datasets demonstrated that no individual model consistently outperformed others; instead, the top-performing algorithm varied substantially between datasets, underscoring the pervasive impact of algorithmic bias on classifier performance [55]. To mitigate these limitations, MetaPaCS integrated both stacking and meta-learning strategies. Stacking ensembles leverage the complementary strengths of diverse classifiers to generate a consensus prediction, while meta-learning enables the model to correct the systematic biases present in its base learners. The results suggest that our method improved the accuracy of the subtype predictions by around 1% in the best-performing meta-learning classifier when compared to the best-performing base classifier in the same run. Furthermore, concatenating the filtered expression data with the probability outputs from Block A provides a richer, subtype-informed feature representation for Block B, enabling the meta-learning classifier to simultaneously exploit the strengths of the base models and retain essential transcriptomic signals. As a result, MetaPaCS achieved performance comparable to or exceeding that of the best base classifier while offering improved robustness across datasets. From the different tests and measured performance of the meta-learner, we determined the optimal model for MetaPaCS to have KNN and QDA as the base classifiers with XGB as the Meta-learner due to its consistently high performance. To evaluate whether calculating gene variability using the complete cohort materially influenced model performance, we repeated the analysis using fold-specific gene filtering. In each iteration, mean expression and CV^2^ were calculated using only the training samples before application to the held-out sample. The revised analysis produced nearly identical results to the MetaPaCS results, including unchanged accuracy (0.9167) and lower precision, recall, *F*1-score, G-measure, and Jaccard index (Figure S6). These findings suggested that the reported predictive performance was robust to fold-specific feature filtering and was not materially driven by inclusion of the held-out sample during CV^2^ calculation. Previous methodological evaluations in small-sample, high-dimensional genomic settings have demonstrated that LOOCV can achieve relatively low bias and mean squared error for prediction error estimation [56]. Additionally, Fırat et al. developed a transcriptomic classifier using 78 subjects and 8815 candidate genes and evaluated its performance using nested cross-validation [57]. Similarly, Shabanian et al. investigated transcriptomic classification in microcohorts of fewer than 40 subjects with approximately 25 000 initial transcriptomic measurements, using dimensionality reduction and repeated resampling to reduce the risk of overfitting [58]. To further assess model robustness, our MetaPaCS framework was evaluated by randomly holding out 20% of samples as an external test set that was never used for feature selection or hyperparameter tuning and the remaining data as the training set. All hyperparameters were tuned only on the remaining 80% training data using cross-validation. The final model was retrained on the full training set and evaluated on the held-out test set. This process was repeated five times. The results suggested that MetaPaCS achieved a mean accuracy of 0.9167, *F*1-score of 0.9173, MCC of 0.8889, and AUC of 0.9852 (Figure S7). These results were comparable to the original LOOCV estimates, indicating that MetaPaCS performance remained stable across different stratified data partitions under the nested validation framework.

In terms of visualization capabilities, MetaPaCS provided a more informative representation of PaC transcriptomes than raw expression data. Conventional dimensionality reduction methods applied directly to the original matrix produced substantial subtype overlap, reflecting the complexity of the data and the inability of unsupervised embeddings to recover subtype structure. In contrast, applying these methods to the MetaPaCS-derived embedding yielded markedly improved separation, indicating that the model captures discriminative signals not apparent in the raw features. Incorporating OH-encoded subtype probabilities further enhanced cluster distinction, improving the interpretability of the visual space. These findings highlight the value of MetaPaCS in generating subtype-aware representations that enable clearer and more meaningful visualization of PaC heterogeneity. DGE analysis of the four subtypes also revealed a variety of important, uniquely upregulated genes that are essential to take into account when giving treatment to a patient with PaC. For example, progenitor displays an upregulation in a gene responsible for the metabolic creation of glucose (G6PC2) and ADEX displays an upregulation in a gene responsible for the digestion of starch (AMY2A), which both serve to heighten blood sugar levels long term. This high blood sugar not only elevates the risk of type 2 diabetes but additionally may encourage cancer cell proliferation with the more easily available energy source. Another example is the upregulated genes in the squamous and immunogenic subtypes, which both influence the cancer characteristics in a way that makes treatment differ from progenitor or ADEX. Squamous, the most lethal subtype, shows many upregulated genes that are also found in other more lethal cancers such as MIR205HG, which is associated with migration and proliferation, and others like KRT6C, which are found to be upregulated in lung adenocarcinoma. These genes likely influence the way a squamous subtype tumor would spread and metastasize, making it an important consideration for how treatment should be carried out. Immunogenic is similar, as the upregulated immunoglobin genes create an immunosuppressant microenvironment, which is important to take into consideration for immunotherapy-based treatments.

In addition, our GO pathway analysis found that each subtype showed upregulation in distinct pathways that lead to the unique characteristics of each subtype. The squamous subtype, for example, showed a high number of upregulated genes in pathways related to skin/dermis development and cellular structure, which would possibly result in a different tumor structure and different metastatic characteristics of tumors in this subtype. Additionally, four of the 15 most upregulated GO pathways in the squamous subtype were found to play a part in kidney development, possibly serving as a driving factor in some of the unique characteristics seen in this subtype. The immune subtype, meanwhile, shows upregulation in a multitude of immune response pathways, which was expected based on its characteristics and previous research. The progenitor and ADEX subtypes are the most similar of the four subtypes, with both having upregulation in pathways meant mostly for the transport and localization of hormones and proteins. The main difference between the two is the upregulation in digestion and insulin secretion pathways seen in the progenitor subtype.

Pathway analysis also revealed further medical considerations that should be taken for those with specific subtypes. Notably, both progenitor and ADEX show upregulation in genes that impact the metabolism of drugs used for diabetes treatment. When combined with the impact on different metabolic genes in the cancer itself, it may lead to as of yet unknown impact on treatment. In addition, all subtypes except immunogenic show an impact on a wide variety of different drugs, with the medications themselves being involved in the treatment of everything from other cancers to heart disease and autoimmune disorders. Proper subtype diagnosis may allow for better monitoring and allocation of drugs for treating diseases co-occurring with the particular case of PaC. To further evaluate the biological validity of the MetaPaCS subtype predictions, differential expression, GO enrichment, and drug–disease association analyses were repeated using the MetaPaCS out-of-fold predicted labels and compared with those obtained using the original Bailey subtype annotations. Overall, the two sets of analyses showed a high degree of concordance in subtype-specific differentially expressed genes, enriched GO pathways, and drug–disease associations (Figures S8–S10). These findings demonstrate that the MetaPaCS-predicted subtypes largely reproduce the biological characteristics associated with the reference subtype annotations, providing complementary biological validation of the model’s predictive ability.

While the results are promising, more research is needed to further optimize and evaluate MetaPaCS before it sees any major use in the medical field. Further research would include a far larger and more diverse dataset to obtain a clearer image of both the subtypes and the contribution the stacking aspect of the model makes to subtype prediction. In summary, MetaPaCS provides a robust and flexible framework for PaC subtype classification by integrating diverse predictive signals into a unified, subtype-aware representation. We believe that MetaPaCS will enhance downstream risk stratification for PaC and contribute to the design of more personalized treatment strategies.

## 5 | Conclusion

In this article, we introduced MetaPaCS, a high-performing ensemble meta-learning method for the classification of PaC into subtypes. Our method was based on a stacking ensemble, using the probability predictions of two base classifiers, including KNN and QDA, concatenated to the original input data as input for a new XGBoost meta-learning classifier. MetaPaCS performed better than the evaluated individual classifiers for PaC subtyping across several performance measures. These findings support the feasibility of integrating complementary classifier outputs with transcriptomic features for PaC subtype classification. Future research aims to make use of a much larger PaC dataset with the inclusion of multi-omics data integration. Given adequate data, this model’s framework may be extensible to subtype prediction in other cancer subtypes as well.

## Supplementary Material

supplementary material

Additional supporting information can be found online in the Supporting Information section.

## Figures and Tables

**FIGURE 1 |:**
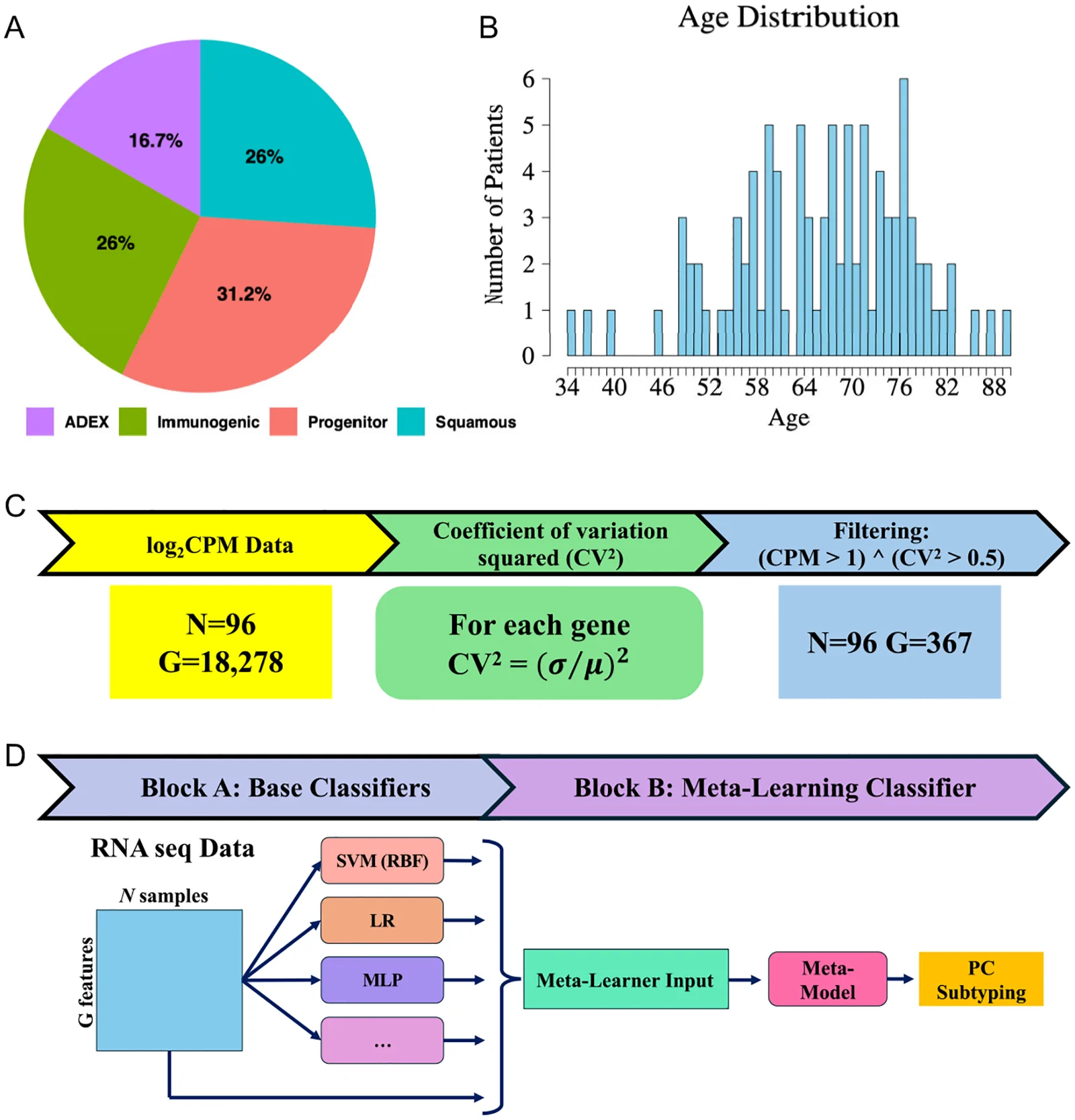
Overview of the MetaPaCS framework. (A) Distribution of subtypes within the dataset. (B) Age demographics of the patient cohort. (C) The original log_2_(CPM) expression values were transformed to log_2_(CPM + 1). Genes were retained based on mean expression >1 and CV^2^ >0.5. (D) MetaPaCS framework optimization workflow. Different subsets drawn from 10 candidate ML algorithms were evaluated in Block A, while candidate algorithms were also assessed as the Block-B meta-learner.

**FIGURE 2 |:**
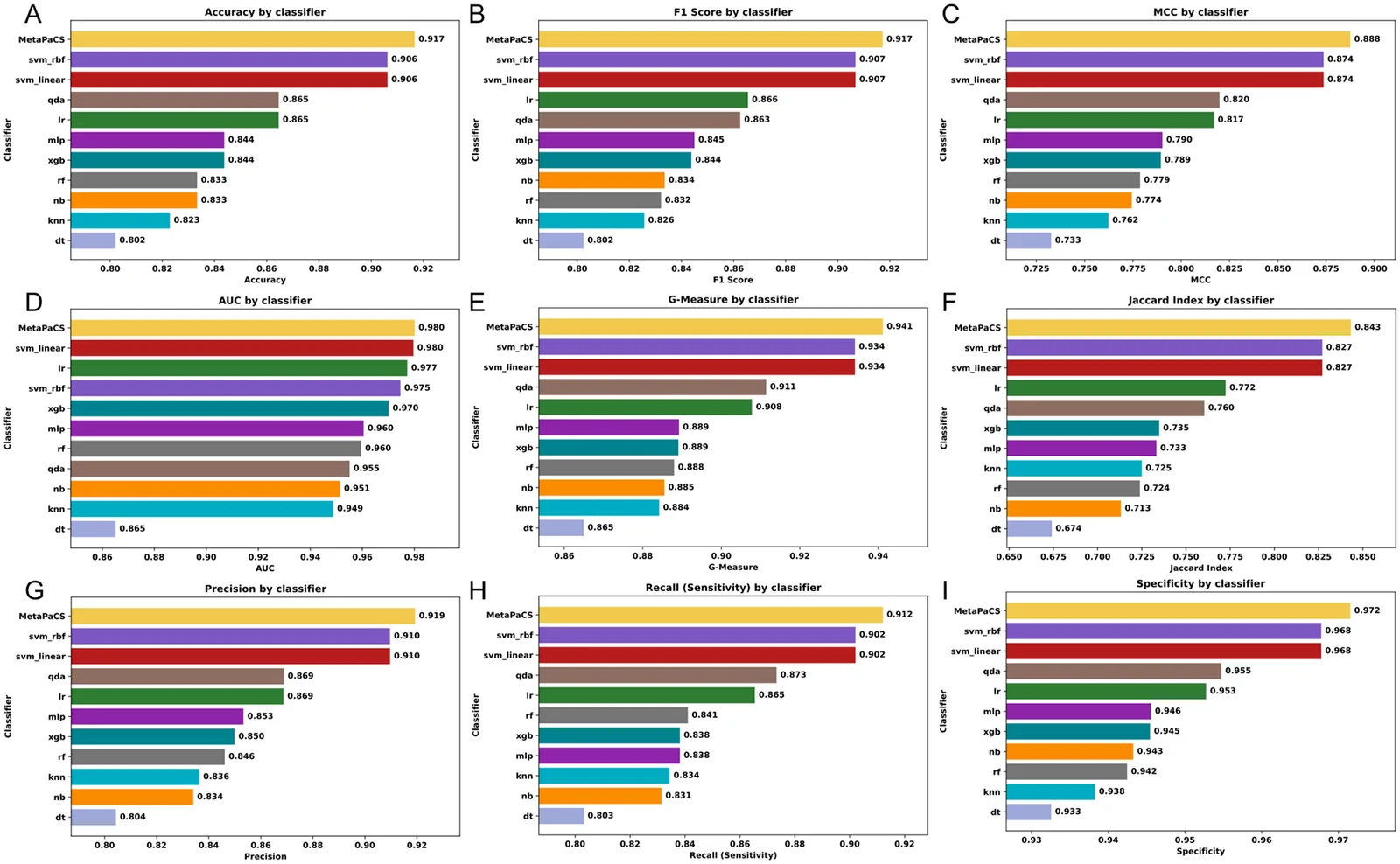
Performance comparison between all base classifiers and MetaPaCS. The *y*-axis denotes the individual base classifiers and MetaPaCS, while the *x*-axis represents the value for each evaluation metric. Subfigures display results for: (A) accuracy, (B) *F1*-score, (C) MCC, (D) area under the curve (AUC), (E) *G*-measure, (F) Jaccard index, (G) precision, (H) recall, and (I) specificity.

**FIGURE 3 |:**
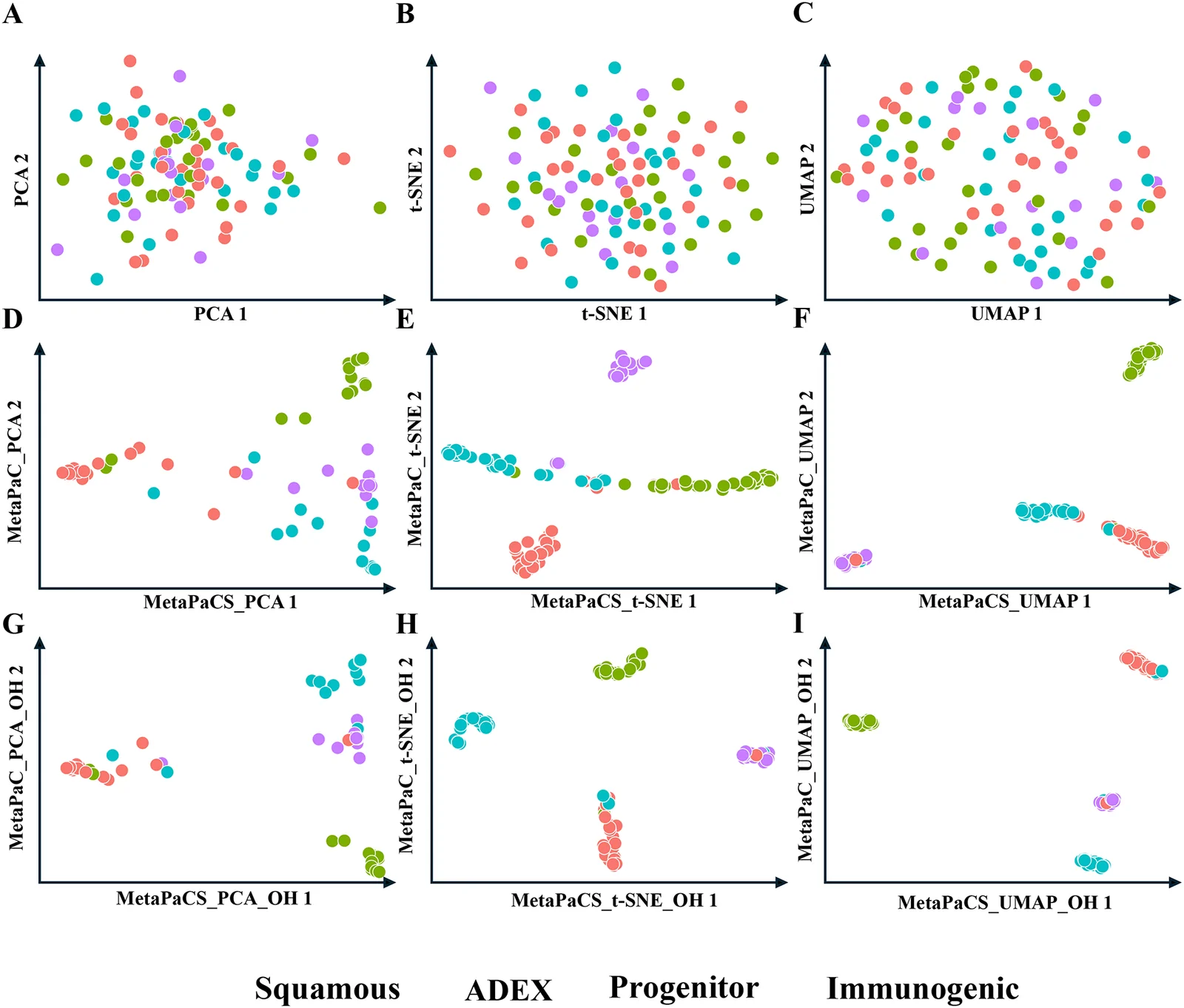
Comparative analysis of dimensionality reduction techniques on filtered gene expression data and corresponding subtype prediction probabilities. Each point represents a sample, colored according to its true subtype. Rows illustrate different strategies: (A–C) the original filtered data displayed with PCA, t-SNE, and UMAP, (D–F) subtype prediction probabilities from MetaPaCS displayed with PCA (MetaPaCS_PCA), t-SNE (MetaPaCS_t-SNE), and UMAP (MetaPaCS_UMAP), and (G–I) subtype prediction probabilities from MetaPaCS concatenated with OH-encoded (OH) results displayed with PCA (MetaPaCS_PCA_OH), t-SNE (MetaPaCS_t-SNE_OH), and UMAP (MetaPaCS_UMAP_OH). Columns represent the dimensionality reduction technique applied: PCA (V1) (A,D,G), t-SNE (V2) (B,E,H), and UMAP (V3) (C,F,I). Panels G–I were designed as a predicted subtype-aware visualization of the MetaPaCS decision space. For each sample, the subtype probability scores and predicted subtype assignment were generated out-of-fold under LOOCV by a model trained without that sample. The one-hot vector was constructed exclusively from the out-of-fold predicted subtype assignment and concatenated with the corresponding feature matrix before PCA, t-SNE, and UMAP were applied.

**FIGURE 4 |:**
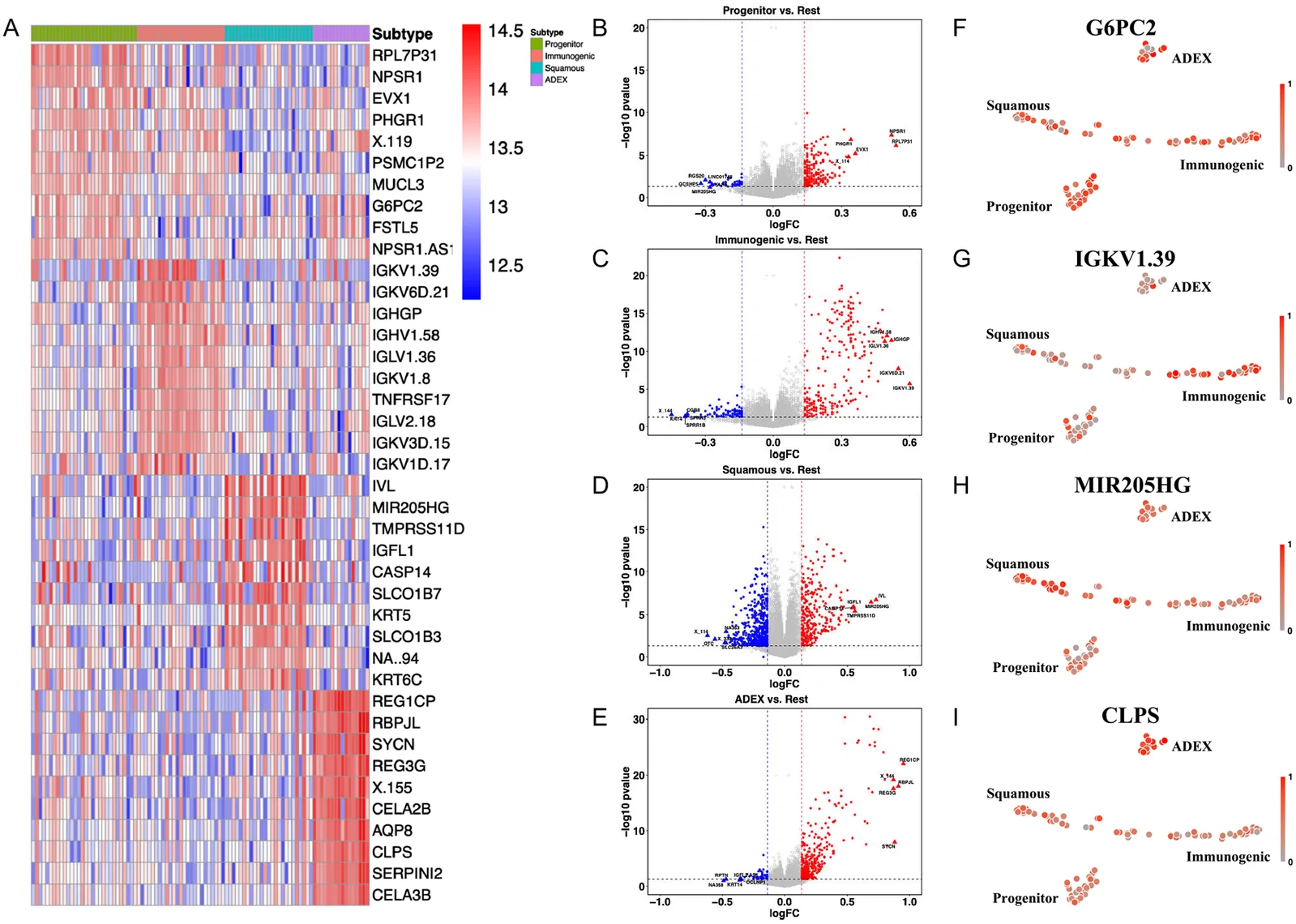
Subtype-specific DGE analysis and relative expression visualization. (A) Heatmap illustrating the top 10 upregulated genes for each subtype in a one-versus-rest comparison. (B–E) Volcano plots highlighting significantly upand downregulated genes (logFC > 1.5 or <−1.5, and *p*-value < 0.05) for: (B) Progenitor, (C) immunogenic, (D) Squamous, and (E) ADEX subtypes. The five genes with the highest and lowest logFC values are labeled. (F–I) t-SNE plots showing the relative expression (red intensity) of a key upregulated gene within each respective subtype: (F) Progenitor, (G) immunogenic, (H) Squamous, and (I) ADEX. Differential expression was assessed separately for each subtype using one-versus-rest comparisons based on empirical Bayes-moderated statistical test which was implemented in *limma* package in R. *P*-values were adjusted for multiple comparisons using the Benjamini–Hochberg method. Genes with logFC > 1.5 and adjusted *P* < 0.05 were considered significantly differentially expressed. The cohort included 16 ADEX, 25 immunogenic, 30 progenitor, and 25 squamous samples.

**FIGURE 5 |:**
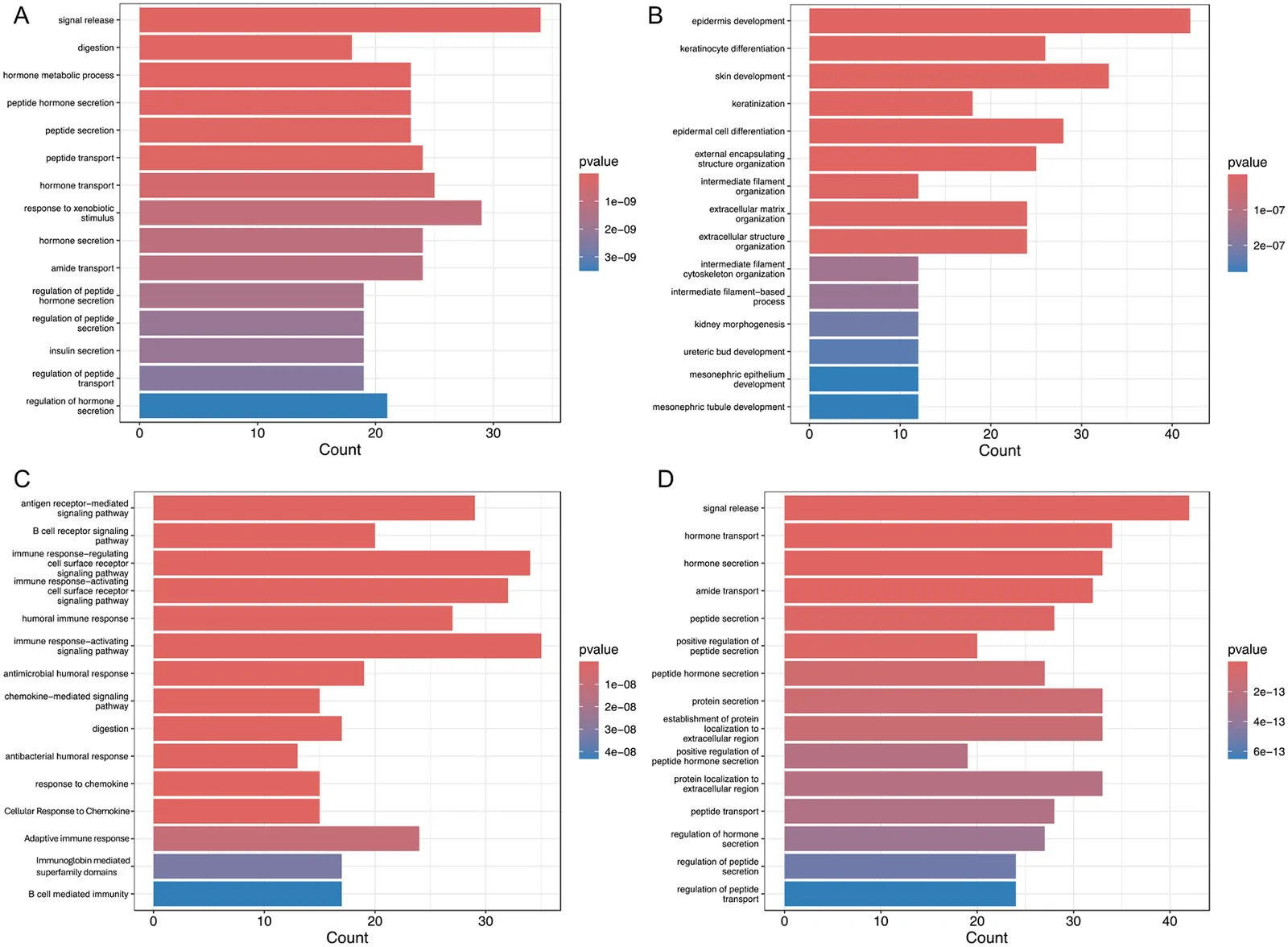
Prominent GO pathways associated with upregulated genes in each subtype. Bar plots illustrating the 15 most significant GO pathways enriched among the top 500 upregulated genes for: (A) progenitor, (B) squamous, (C) immunogenic, and (D) ADEX subtypes. GO enrichment analysis was performed using *clusterProfiler* package in R for the top 500 upregulated genes from each one-versus-rest comparison. *P*-values were adjusted using the Benjamini–Hochberg method. The 15 most significantly enriched terms are presented for each subtype.

**FIGURE 6 |:**
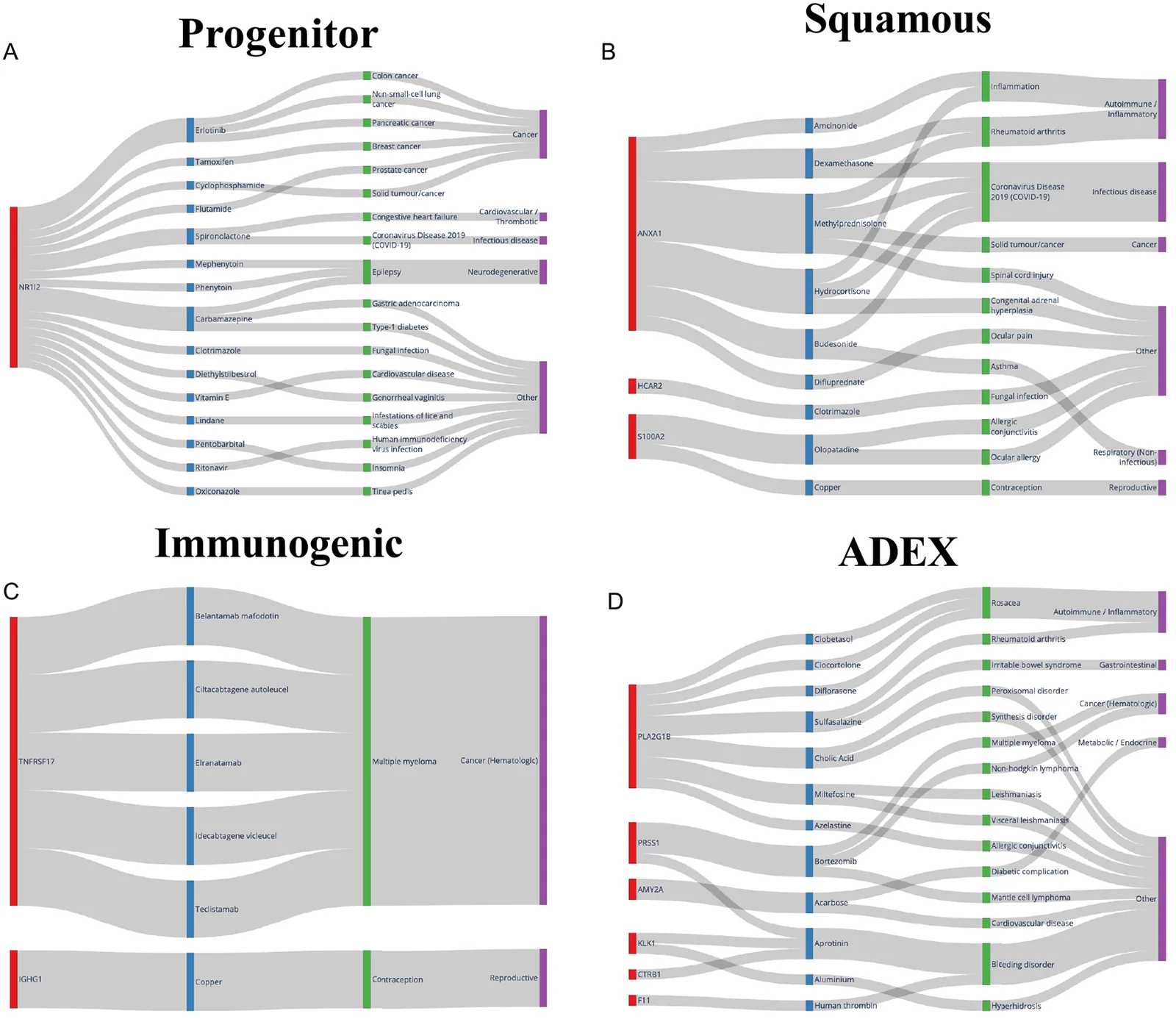
Drug and disease pathway associations for differentially expressed genes in each subtype. Sankey diagrams illustrating connections between highly differentially expressed genes and associated drug and disease pathways from the DrugBank database and TTD for: (A) progenitor, (B) squamous, (C) immunogenic, and (D) ADEX subtypes.

## Data Availability

All code used for MetaPaCS is publicly available and can be found on GitHub at https://github.com/wan-mlab/MetaPaCS.

## Data Availability Statement

All data used in this study are publicly available in the corresponding references mentioned in the manuscript.
