# Supplementary material for: MetaPaCS: A Novel Meta-Learning Framework for Pancreatic Cancer Subtype Identification
Source: Adv Intell Syst. Author manuscript; Available in PMC 2026 Nov 7. (PMC13742208; doi:10.1002/aisy.70576)
Supplement: Supplementary file 1 [file NIHMS2217699-supplement-supplementary_material.pdf]

Block A

Classifier	Parameters
MLP (Multi-layer Perceptron)	activation: "relu"<br>alpha: 0.0001<br>learning_rate: "constant"<br>max_iter: 210<br>batch_size: 8<br>solver: "adam"<br>hidden_layer_sizes: (100, 100)
LR (Logistic Regression)	max_iter: 700
NB (Naïve Bayes)	default
RF (Random Forest)	default
SVM (Linear)	default
SVM (RBF)	default
XGB (XGBoost)	n_estimators: 300<br>use_label_encoder: False<br>eval_metric: 'logloss'
QDA (Quadratic Discriminant Analysis)	default
KNN (K Nearest Neighbors)	default
DT (Decision Tree)	default

Block B

Classifier	Parameters
MLP (Multi-layer Perceptron)	activation: "relu"<br>alpha: 0.0001<br>learning_rate: "constant"<br>max_iter: 210<br>batch_size: 8<br>solver: "adam"<br>hidden_layer_sizes: (100, 100)
LR (Logistic Regression)	default
NB (Naïve Bayes)	default
RF (Random Forest)	default
SVM (Linear)	default
SVM (RBF)	default
XGB (XGBoost)	n_estimators: 300<br>use_label_encoder: False<br>eval_metric: 'logloss'
QDA (Quadratic Discriminant Analysis)	default
KNN (K Nearest Neighbors)	default
DT (Decision Tree)	default

Supplementary Table 1. All tested Base and Meta-learners

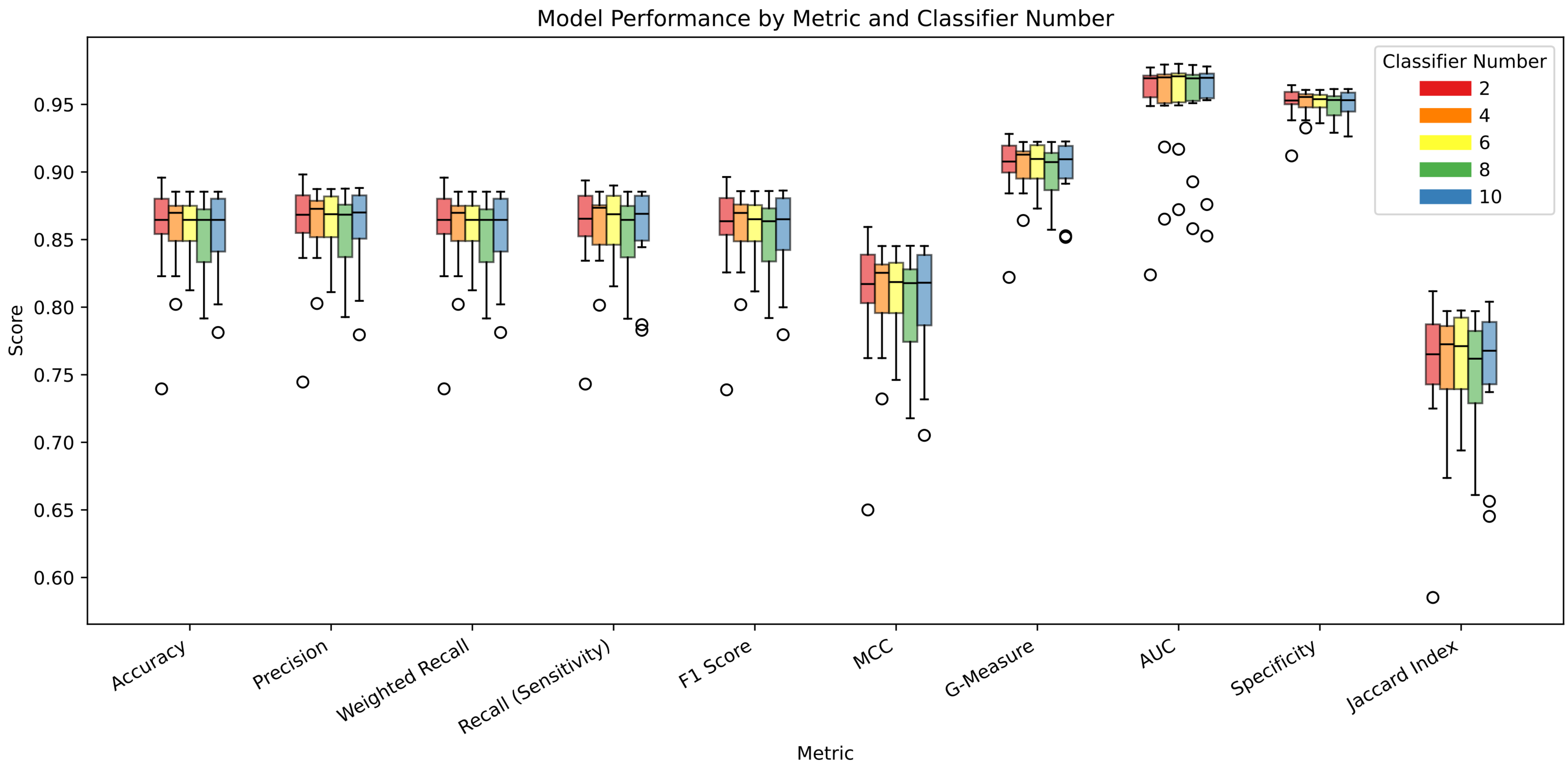

Supplementary Fig. S1 An evaluation of the effect of different numbers of base classifiers on meta-model performance. The X axis shows which metric the scores belong to while the Y axis shows the score of all tested meta-learners for the metric being evaluated.

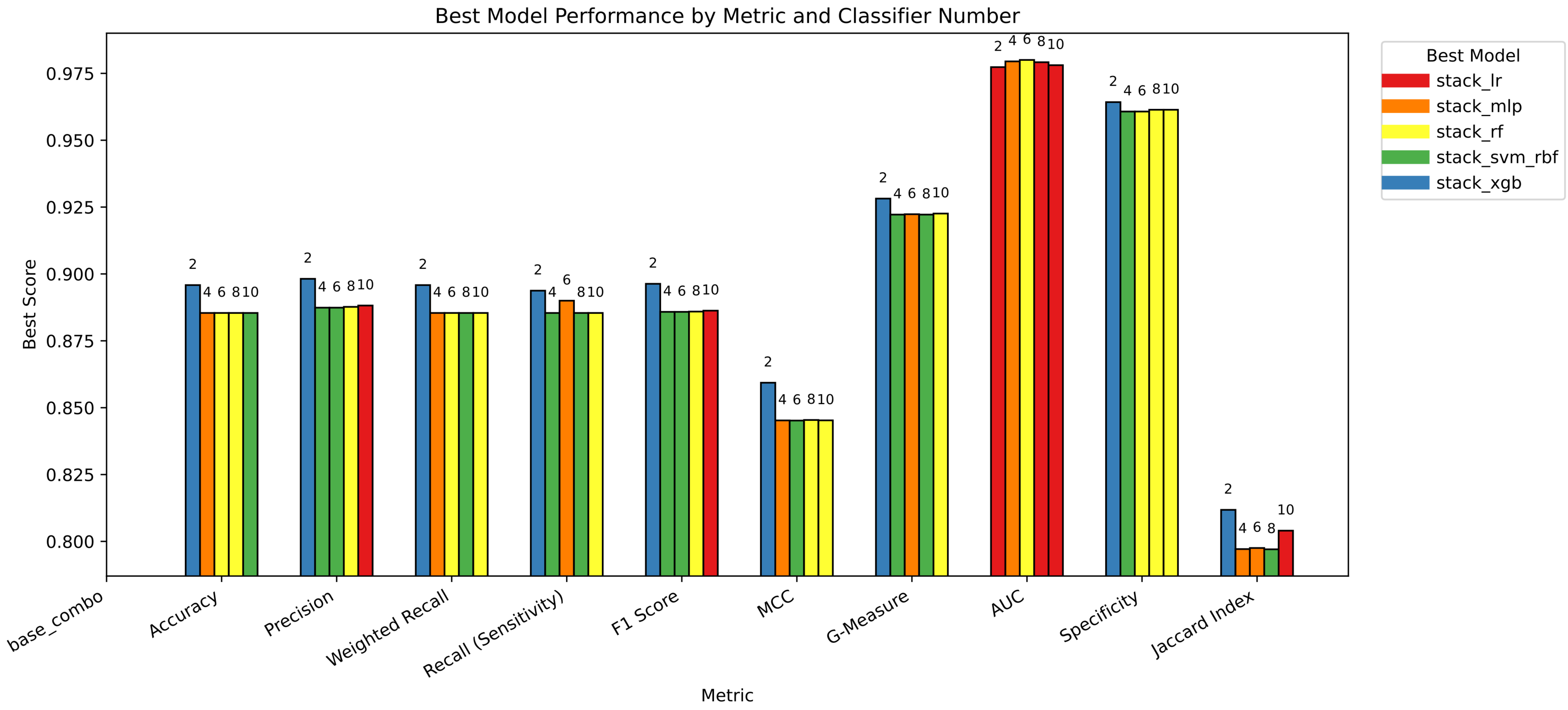

Supplementary Fig. S2 an evaluation of the best performing meta-learner for different numbers of base classifiers. The X axis shows which metric the scores belong to while the Y axis shows the best meta-learner score for the metric being evaluated.

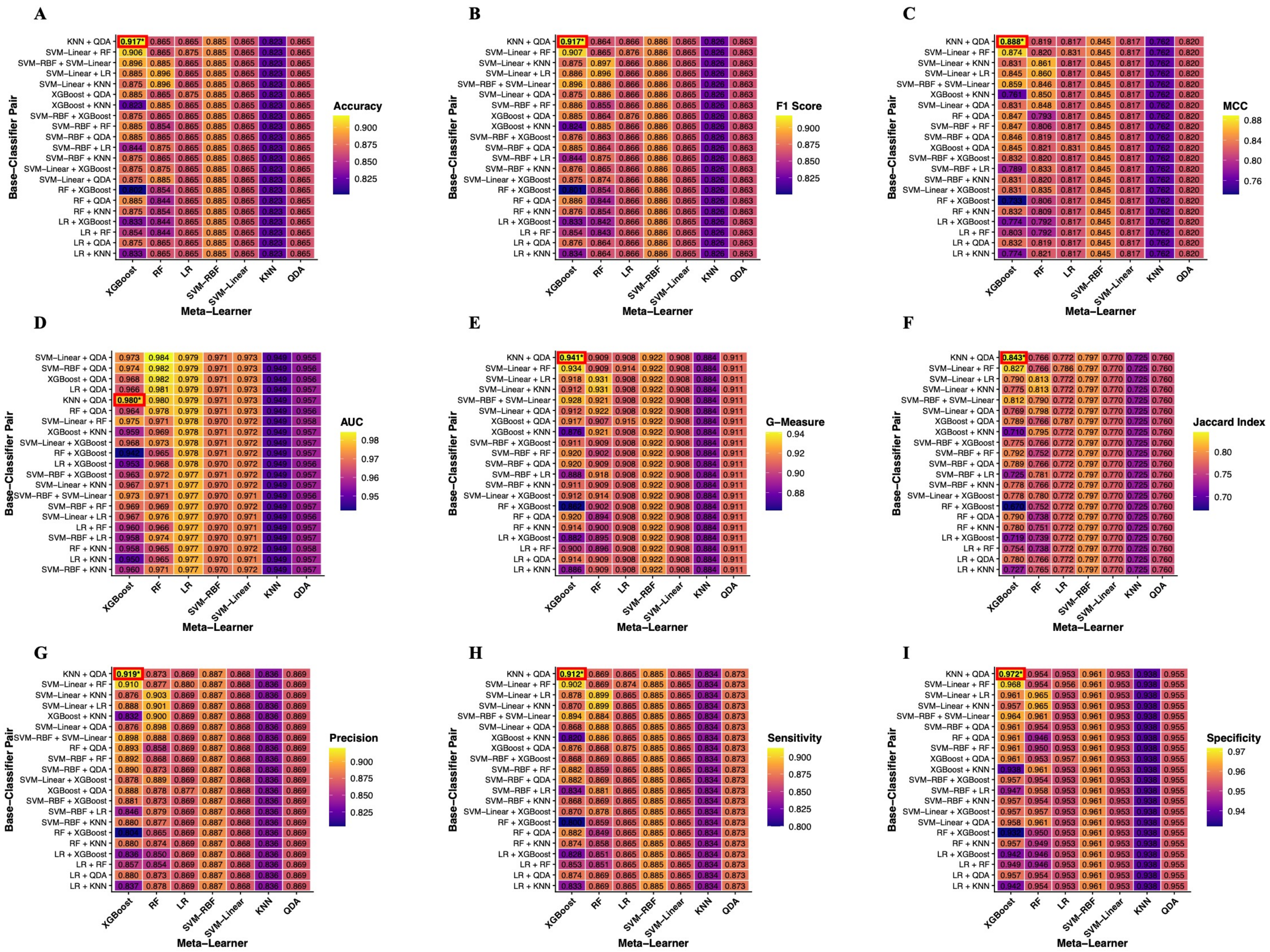

Supplementary Fig. S3 Performance comparison of all pairwise base-classifier and meta-learner configurations for pancreatic cancer subtyping. Heatmaps show the performance of 147 model configurations generated from all 21 pairwise combinations of the seven top-ranked base classifiers and seven candidate meta-learners. Performance was evaluated using (**A**) accuracy, (**B**) F1-score, (**C**) Matthews correlation coefficient (MCC), (**D**) area under the receiver operating characteristic curve (AUC), (**E**) G-measure, (**F**) Jaccard index, (**G**) precision, (**H**) sensitivity, and (**I**) specificity. Each cell represents one base-classifier pair and meta-learner configuration. The selected configuration, consisting of KNN and QDA as the base learners and XGBoost as the meta-learner, is highlighted by the red outline and asterisk. *, Selected MetaPaCS framework: KNN + QDA / XGBoost.

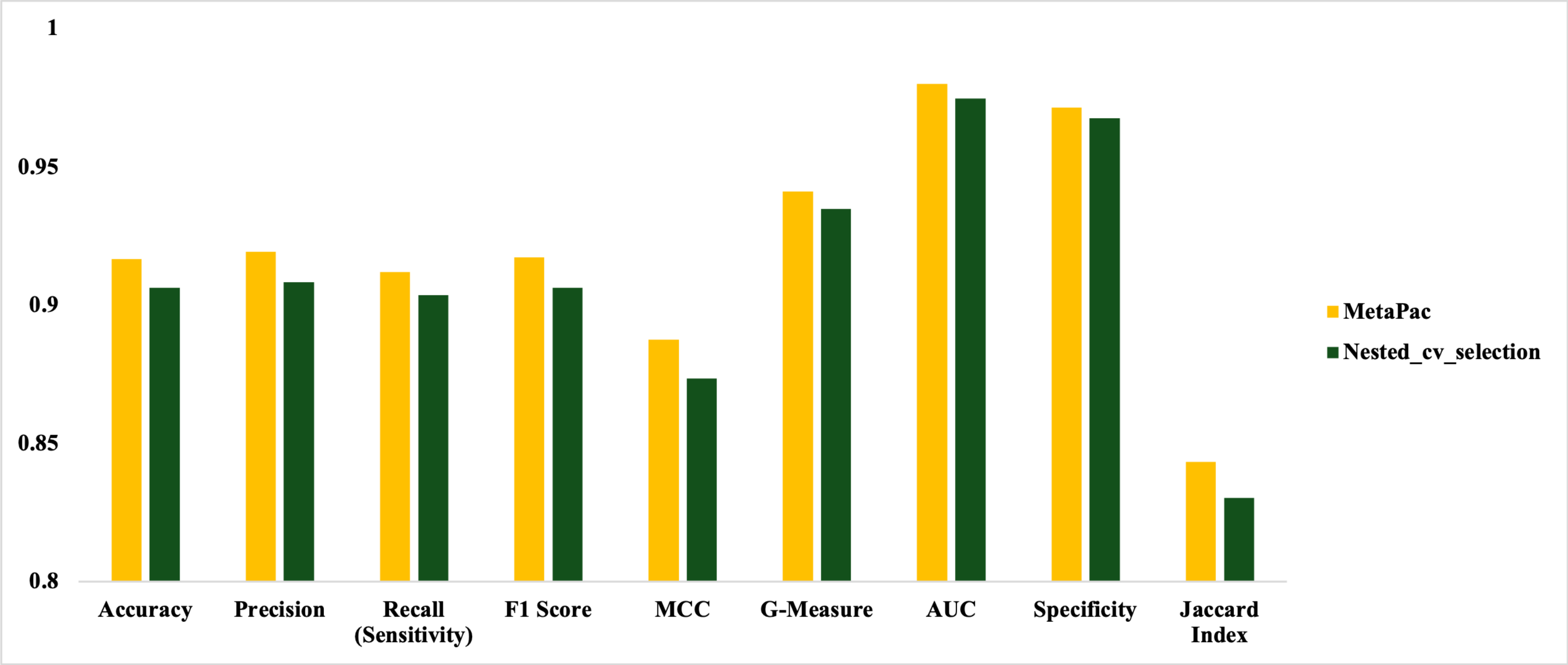

Supplementary Fig. S4 Performance comparison between MetaPaCS and the best-performing alternative framework identified through nested cross-validation. The selected MetaPaCS framework comprised KNN and QDA as the Block A classifiers and XGBoost as the Block B meta-learner. The nested cross-validation analysis evaluated 147 candidate frameworks and identified LR–QDA/XGBoost as the best-performing alternative. Performance was assessed across all pancreatic cancer samples using accuracy, precision, sensitivity, F1 score, MCC, G-measure, AUC, specificity, and Jaccard index.

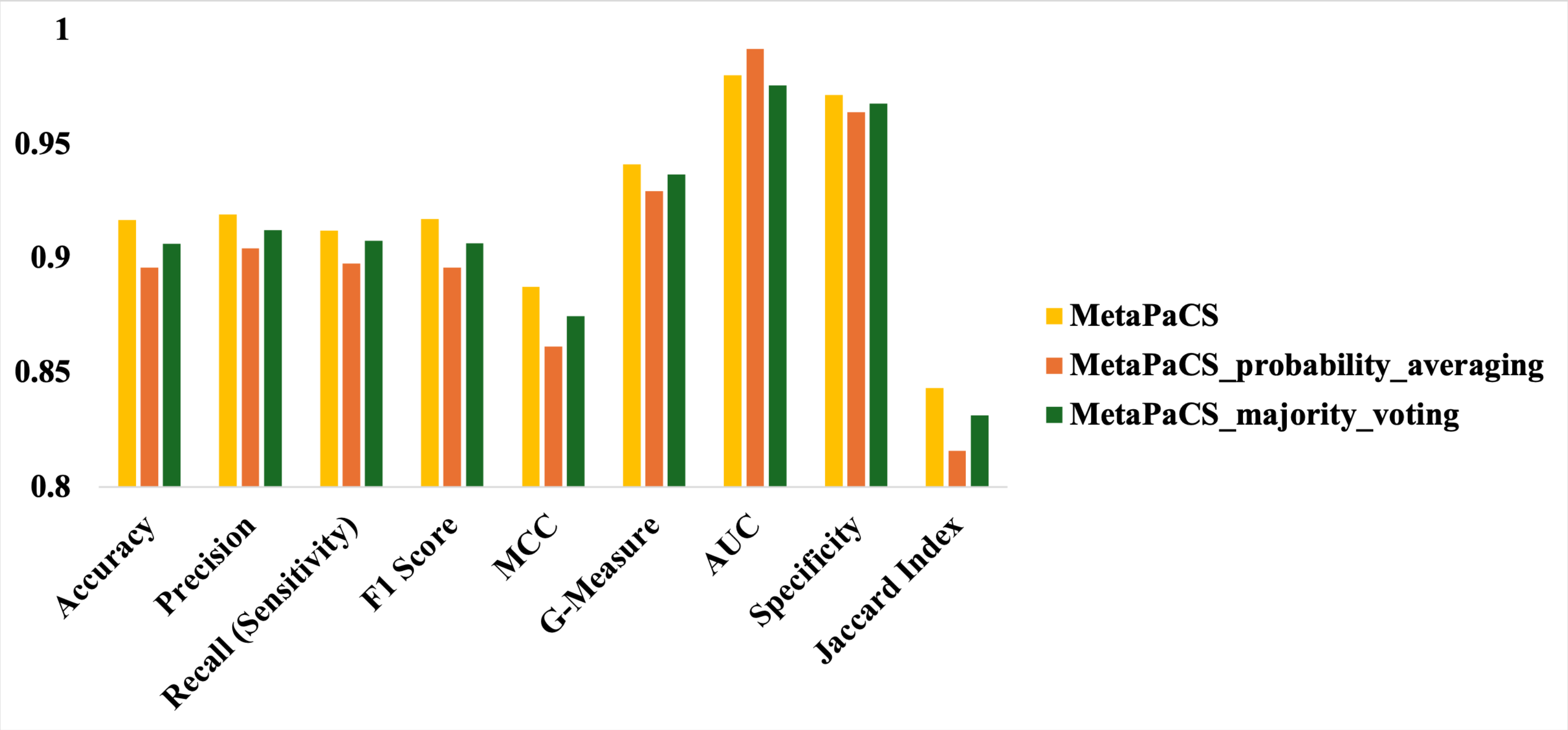

Supplementary Fig. S5 Comparison of MetaPaCS with simple ensemble baselines for PaC subtyping. Performance of MetaPaCS, probability averaging, and majority voting using KNN, QDA, and XGBoost was evaluated on all pancreatic cancer samples using leave-one-out cross-validation. Probability averaging was performed by taking the unweighted mean of the subtype probability vectors generated by the three classifiers, whereas majority voting was based on their predicted subtype labels.

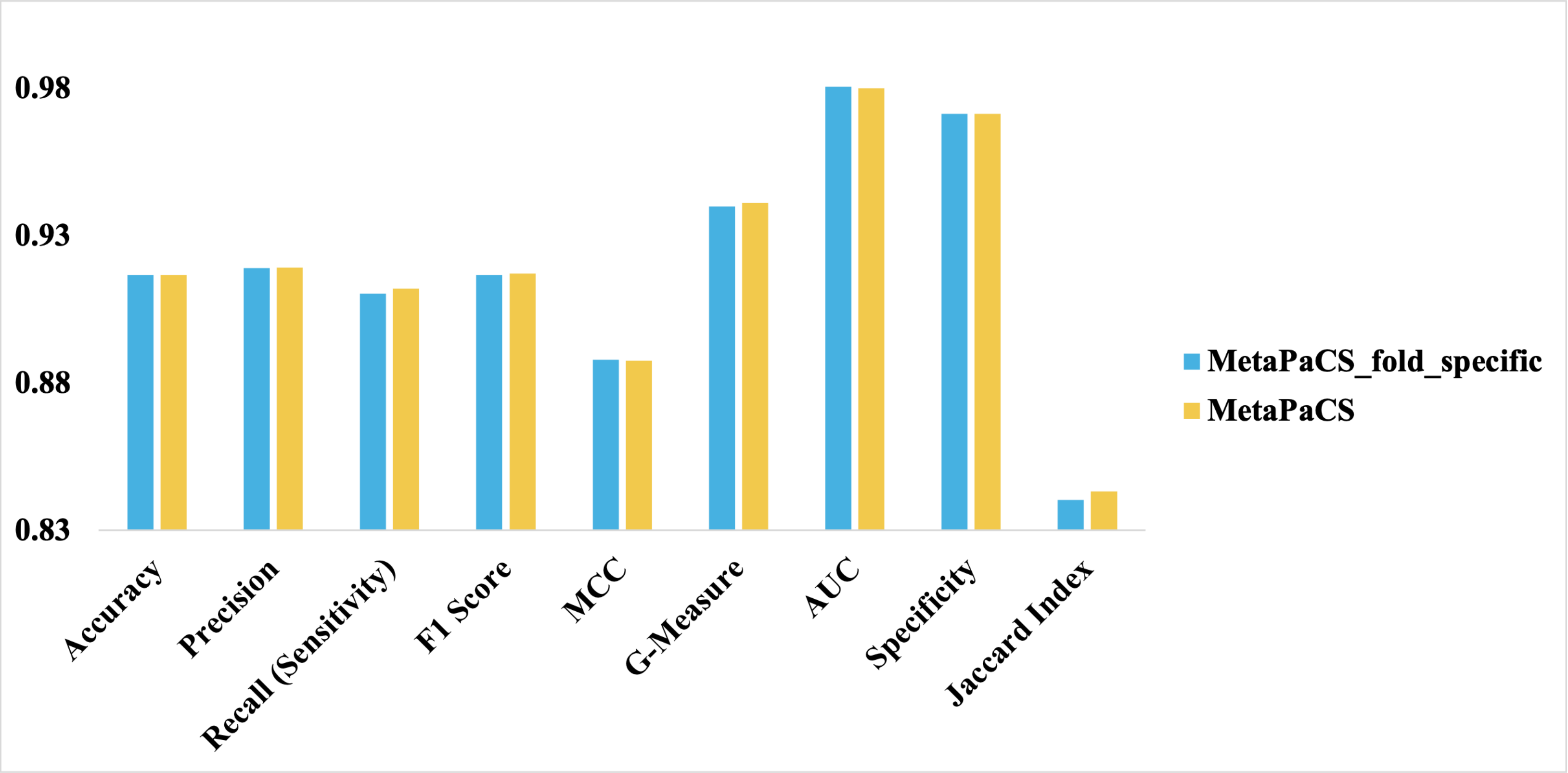

Supplementary Fig. S6 Comparison of MetaPaCS performance using original and fold-specific gene filtering for PaC subtyping. In the fold-specific analysis, gene-wise mean expression and CV^2 were calculated exclusively from the training samples in each LOOCV iteration. Genes with mean $\log_2^{(CPM+1)}$ expression > 1 and CV^2 > 0.5 were retained and subsequently applied to the held-out sample.

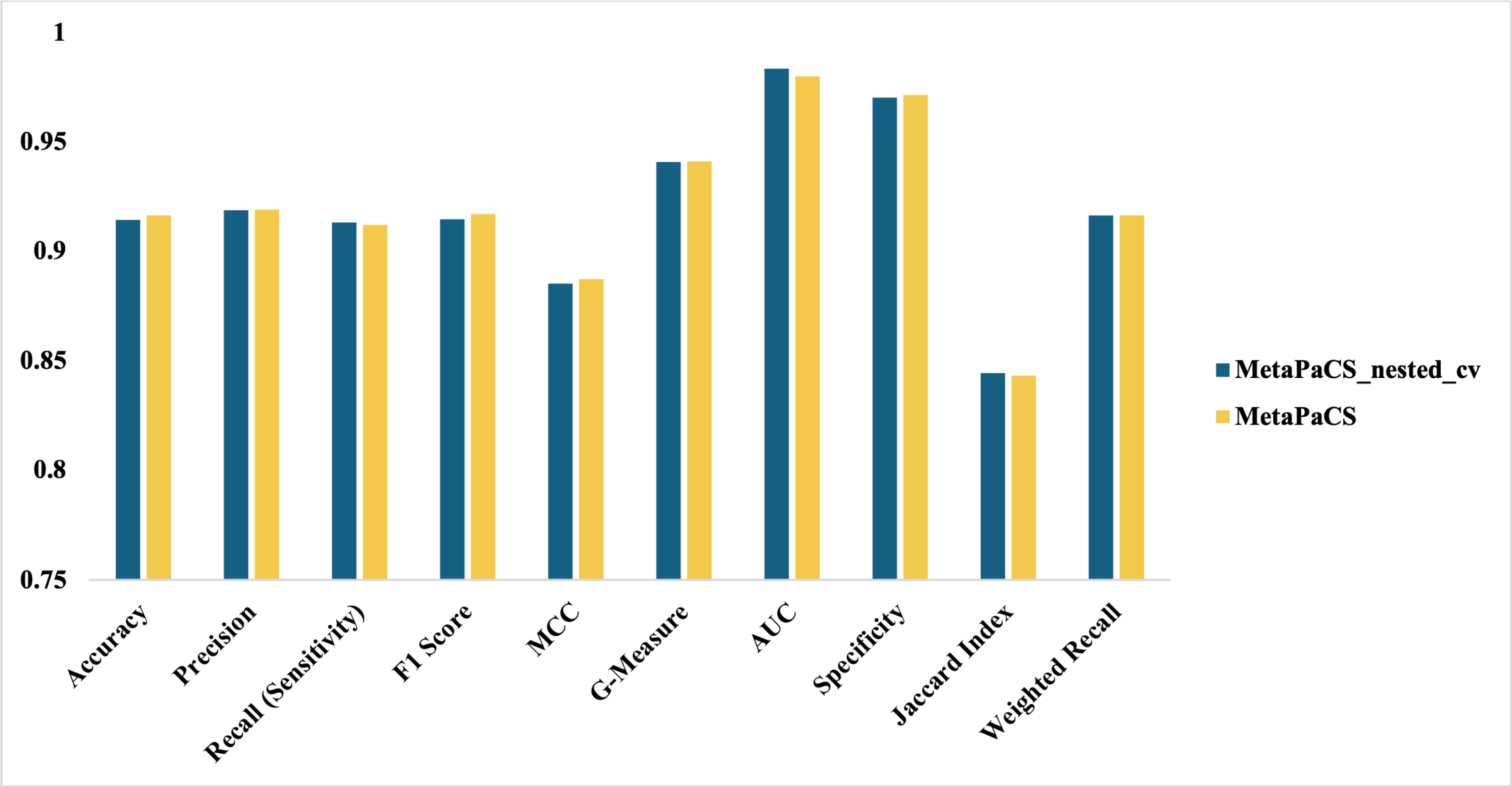

Supplementary Fig. S7 Comparison of MetaPaCS performance under repeated held-out validation and the original LOOCV analysis. The MetaPaCS framework comprised KNN and QDA as the Block A classifiers and XGBoost as the Block B meta-learner. For each validation run, 20% of the samples were randomly held out as an independent test partition, while the remaining 80% were used for model training. Feature selection and hyperparameter tuning were performed exclusively within the training partition, and the final model was evaluated on the held-out samples. This procedure was repeated 20 times using different data partitions. The reported held-out validation values represent the mean across the 20 repetitions. Performance was comparable with the original LOOCV estimates across the evaluated metrics.

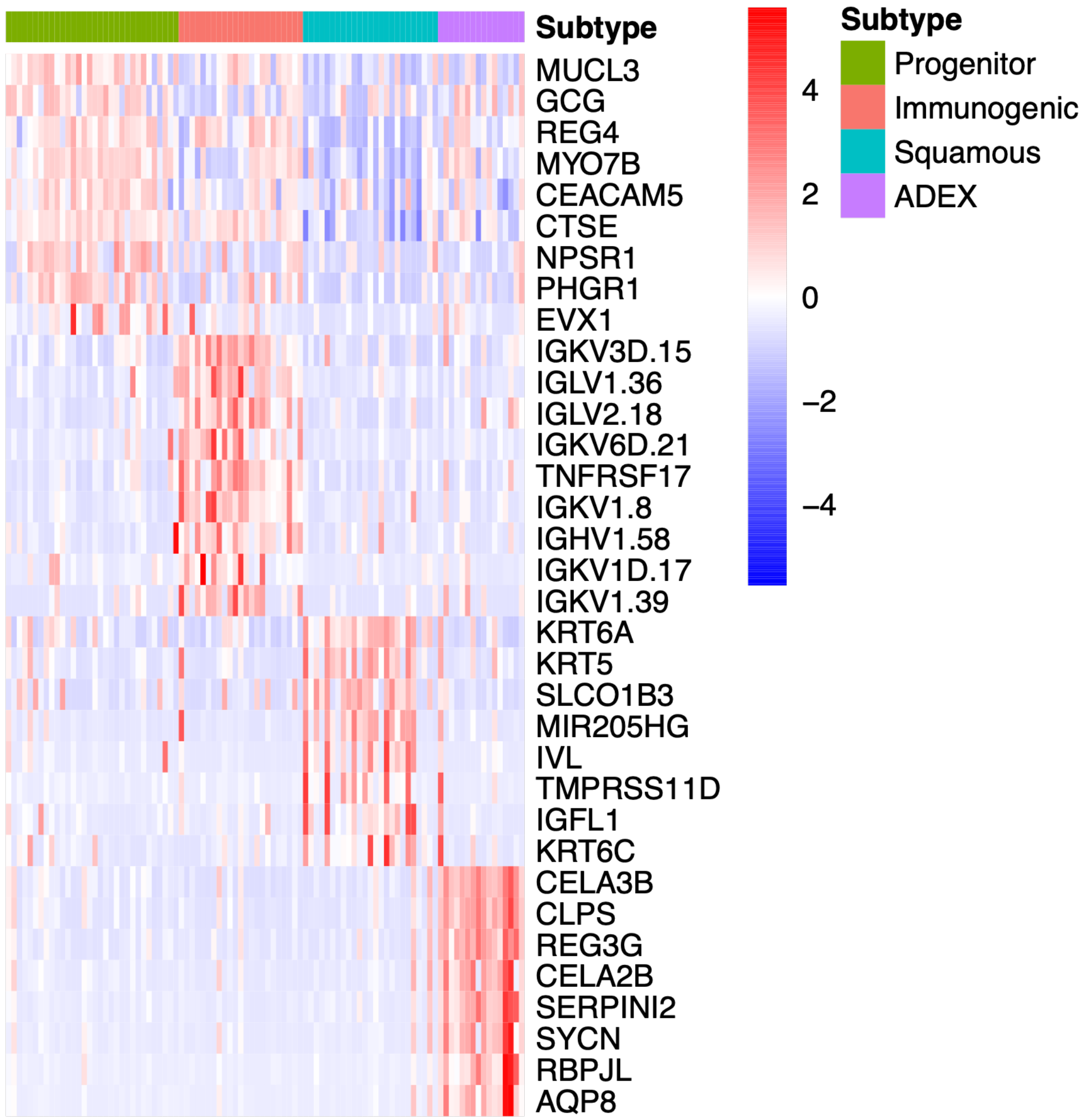

Supplementary Fig. S8 Subtype-specific differential gene expression (DGE) analysis and relative expression visualization. Heatmap illustrating the top ten upregulated genes for each subtype in a one-vs-rest comparison.

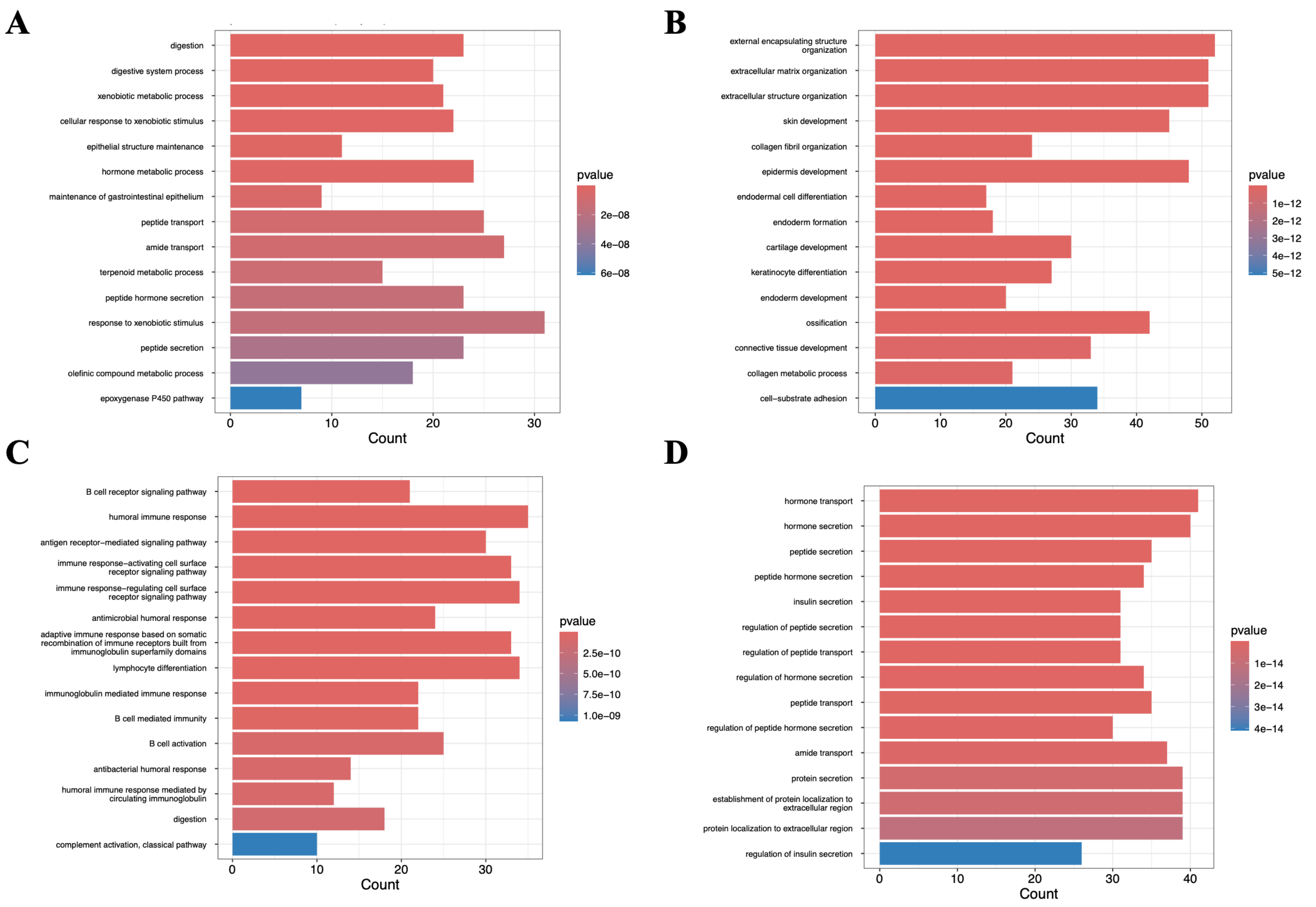

Supplementary Fig. S9 Prominent Gene Ontology (GO) pathways associated with upregulated genes identified using MetaPaCS-predicted subtype labels. Bar plots illustrating the 15 most significant GO pathways enriched among the top 500 upregulated genes for (**A**) Progenitor, (**B**) Squamous, (**C**) Immunogenic, and (**D**) ADEX subtypes. Differential expression was performed using one-versus-rest comparisons based on the MetaPaCS out-of-fold predicted subtype labels. GO enrichment analysis was performed using the clusterProfiler package in R for the top 500 upregulated genes from each subtype. P values were adjusted using the Benjamini–Hochberg method. The 15 most significantly enriched GO terms are presented for each subtype.

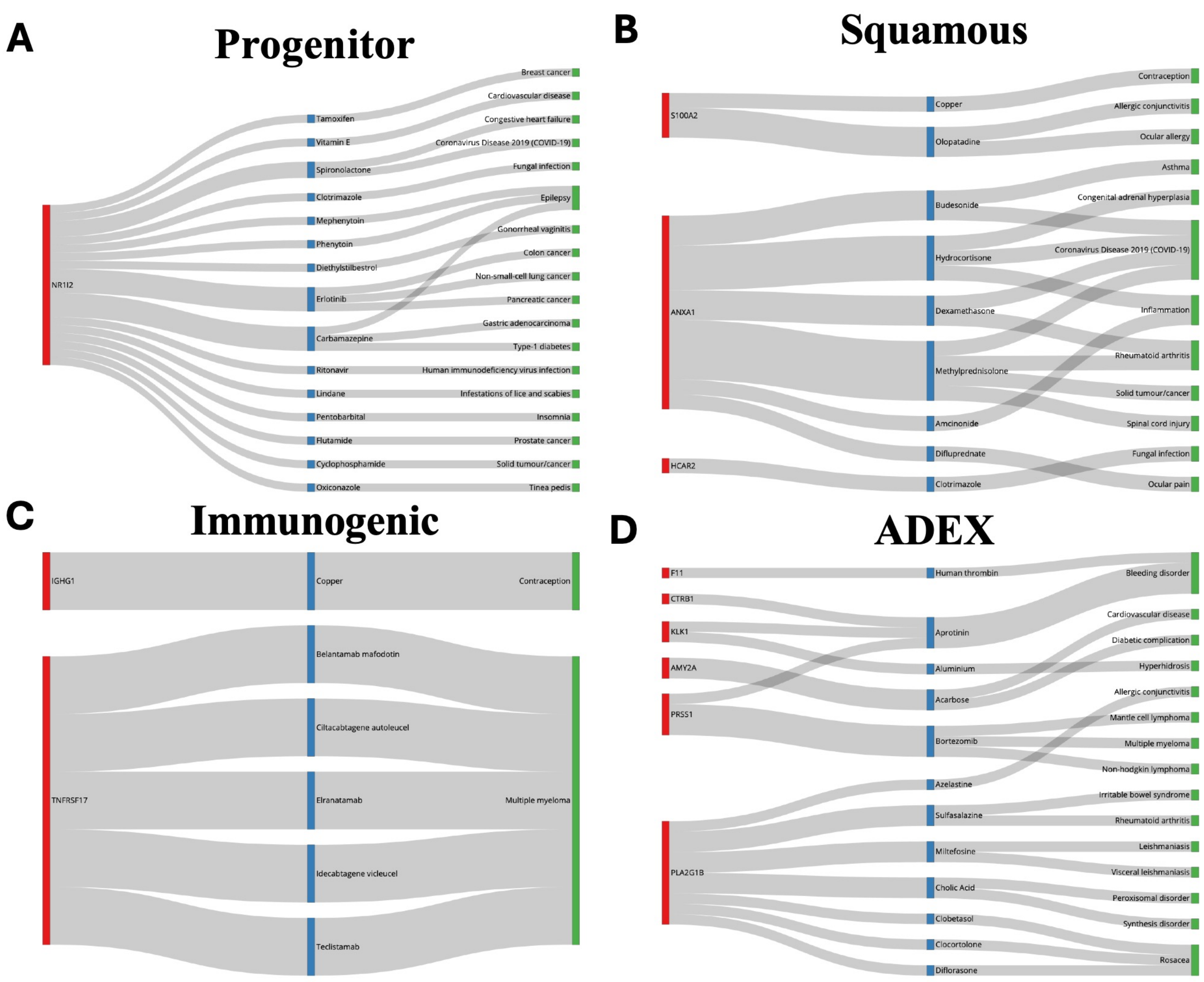

Supplementary Fig. S10 Drug and disease pathway associations for differentially expressed genes identified using MetaPaCS-predicted subtype labels. Sankey diagrams illustrating connections between highly differentially expressed genes and associated drug and disease pathways from the DrugBank and Therapeutic Target Database (TTD) databases for (**A**) Progenitor, (**B**) Squamous, (**C**) Immunogenic, and (**D**) ADEX subtypes. Differentially expressed genes were identified using one-versus-rest comparisons based on the MetaPaCS out-of-fold predicted subtype labels.
